# Gas‐Shearing Fabrication of Multicompartmental Microspheres: A One‐Step and Oil‐Free Approach

**DOI:** 10.1002/advs.201802342

**Published:** 2019-02-28

**Authors:** Guosheng Tang, Ranhua Xiong, Dan Lv, Ronald X. Xu, Kevin Braeckmans, Chaobo Huang, Stefaan C. De Smedt

**Affiliations:** ^1^ College of Chemical Engineering Jiangsu Key Lab of Biomass‐based Green Fuels and Chemicals Nanjing Forestry University (NFU) Nanjing 210037 P. R. China; ^2^ Laboratory of General Biochemistry and Physical Pharmacy Faculty of Pharmaceutical Sciences Ghent University Ottergemsesteenweg 460 9000 Ghent Belgium; ^3^ Department of Precision Machinery and Precision Instrumentation University of Science and Technology of China Hefei 230022 P. R. China; ^4^ Department of Biomedical Engineering The Ohio State University Columbus OH 43210 USA

**Keywords:** biocompatible carriers, gas‐shearing, multicompartmental microspheres, one‐step fabrication, tissue engineering

## Abstract

Multicompartmental microparticles (MCMs) have attracted considerable attention in biomedical engineering and materials sciences, as they can carry multiple materials in the separated phases of a single particle. However, the robust fabrication of monodisperse, highly compartmental MCMs at the micro‐ and nanoscales remains challenging. Here, a simple one‐step and oil‐free process, based on the gas‐flow‐assisted formation of microdroplets (“gas‐shearing”), is established for the scalable production of monodisperse MCMs. By changing the configuration of the needle system and gas flow in the spray ejector device, the oil‐free gas‐shearing process easily allows the design of microparticles consisting of two, four, six, and even eight compartments with a precise control over the properties of each compartment. As oils and surfactants are not used, the gas‐shearing method is highly cytocompatible. The versatile applications of such MCMs are demonstrated by producing a magnetic microrobot and a biocompatible carrier for the coculturing of cells. This research suggests that the oil‐free gas‐shearing strategy is a reliable, scalable, and biofriendly process for producing MCMs that may become attractive materials for biomedical applications.

Multicompartmental microparticles (MCMs) are under investigation for applications such as multidrug delivery systems,^1^ in cell culturing technologies,^2^ for multienzyme tandem reactions,^3^ as micromotors,[[qv: 1f,4]] for multitarget detection,^5^ as multifunctional encoded materials,^6^ etc.^7^ The full potential of MCMs remains to be explored, which is partly due to limitations in the production of MCMs. Several technologies have been developed to fabricate compartmentalized particles, including sputter deposition technology,^8^ using pickering emulsions,^9^ layer‐by‐layer self‐assembly,^10^ using microfluidics,^11^ protonation deprotonation cycling,^12^ electrohydrodynamic cojetting,^13^ and centrifugation‐based methods.[[qv: 2b,5a,14]] Recently, microfluidic technology has been extensively explored to fabricate MCMs, as it allows the highest control over the morphology and complexity of particles.^15^ However, a serious restriction of microfluidic technology often comes when sensitive biological molecules have to be encapsulated in MCMs, since the use of oils, photoinitiators, crosslinkers, surfactants, and UV‐irradiation is inevitably required.[[qv: 11b,c,16]] To overcome these limitations, strenuous efforts including centrifugation‐based methods,[[qv: 2b,14c]] multiplex coaxial flow focusing,^17^ and in‐air microfluidics^18^ have been explored to fabricate MCMs for biomedical purposes. Despite this, fabricating such “biofriendly” MCMs with a super compartmentalized and controllable morphology in a one‐step, green, and high‐throughput process remains challenging.

Inspired by some studies that report on the use of gases to fabricate microcapsules or particles,^19^ we took on the challenge of designing MCMs by gas‐shearing to avoid oils and make use of a homemade coaxial needle system (**Figure** **1**). As shown in this report, MCMs with a size ranging from tens to hundreds of micrometers can be easily obtained and the size can be precisely controlled by adjusting the gas (nitrogen) flow. The production of the MCMs can be scaled up by increasing the flow rate of the polymer solution. Importantly, we can easily fabricate MCMs containing as many as eight compartments (known as “eight‐faced” microspheres). As far as the authors are aware, current technologies are limited to six‐faced microspheres.[[qv: 14c]] The MCMs are produced through a “cytocompatible” oil‐free process, and cells can be encapsulated in the various compartments of the microparticles.

**Figure 1:**
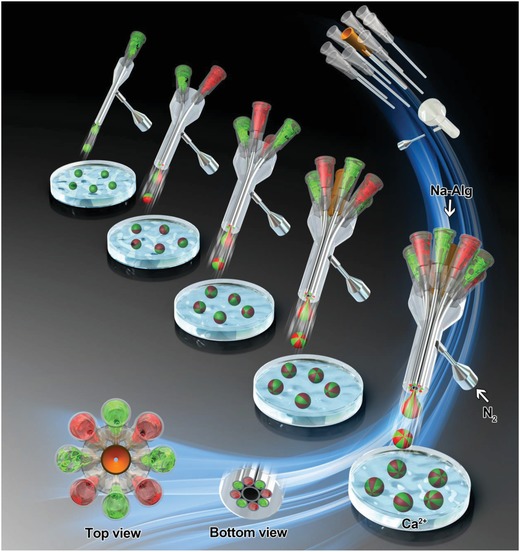
Schematic illustration of the formation of the multifaced microspheres with different spray ejector devices (SEDs) and the assembly of the needle system in the SED‐8 configuration.

The device for fabricating MCMs consists of four major parts (as shown in Figure S1a and Movie S1, Supporting Information): an injection digital pump, a collecting bath, a gas holder, and a homemade coaxial needle system known as a spray ejector device (SED). The SED is the most critical part of the device. As shown in Figure S1, Supporting Information, the liquid‐flow needles are inserted coaxially in a shell. The nitrogen gas is transported through the space between the needle and the shell, generating a shear force allowing the formation of droplets, termed “gas‐shearing” in this study. Based on the number of liquid‐flow needles, we designed the SED‐1, SED‐2, SED‐4, SED‐6, and SED‐8 configurations (containing 1, 2, 4, 6, and 8 needles, respectively). A central holder was added to optimally align the needles coaxially in the SED‐6 and SED‐8 configurations (and to keep the needle system stable).

To evaluate the feasibility of the method, we first fabricated one‐faced microspheres, as schematically shown in **Figure** **2**a and Figure S1a, Supporting Information. The SED‐1 configuration was coupled to the syringe loaded with sodium alginate (Na‐Alg) solution. The flow rate of the polymer solution was controlled by the digital pump. A nitrogen flow (controlled by a rotameter) was used to induce dripping of the Na‐Alg droplets. In this process, the growing droplet experiences two competing forces (assuming the effect of gravity to be neglected): shear forces from the gas pulling the droplet down and forces which arise from the surface tension holding the droplet on the tip. This force balance is given by *ŋ*_gas_ *u*_gas_ *d*_drop_ ∼ *γd*_tip_, where *ŋ*_gas_ is the viscosity of the gas, *u*_gas_ is the mean velocity of the gas, *d*_drop_ is the mean diameter of the droplet, *d*_tip_ is the diameter of the inner needle, and γ is the surface tension.^20^ Initially, the surface tension dominates, though, as the droplet grows the shear force by the nitrogen flow becomes comparable. When the shear force by the nitrogen flow overcomes the resistance force by the surface tension, a droplet is detached from the liquid flow^21^ (Movies S3 and S4, Supporting Information). The CaCl_2_ aqueous solution in the collection bath solidified the liquid droplets into Ca‐Alg microspheres (Movie S2, Supporting Information). Figure 2b shows good agreement between the outcome of the CFD (computational fluid dynamics) simulations for droplet formation and experimental images for a nitrogen flow of 0.4 L min^−1^ and a liquid flow of 0.1 L min^−1^ (Movies S3 and S4, Supporting Information). Furthermore, examples of the velocity field droplet formation are presented in Figure 2b (bottom panel) (Movie S3, Supporting Information) to show the generation of the droplet due to the shear force caused by the velocity gradient. As shown in Figure 2c,d, microspheres with a very low polydispersity can be fabricated. Figure 2e,f clearly reveals that the nitrogen flow significantly dominates the size of the microspheres. Increasing the nitrogen flow results in smaller microspheres. Microspheres with a size between 55 and 1400 µm can be prepared simply by increasing the nitrogen flow from 0.1 to 1 L min^−1^ without any modification to the device. We note that other methods do not allow the production of such a broad size range of particles.[[qv: 2b,22]] In addition to the nitrogen flow, we also assessed the influence of the receiving angle and receiving distance, the space between the needle (core) and the shell, the flow rate of the Na‐Alg solution, and the concentration of the Na‐Alg and CaCl_2_ solutions. Interestingly, as shown in Figure S2, Supporting Information (panel a), pendant and regular microspheres can be obtained using a receiving angle of 0° and 90°, respectively. As expected, using devices with a smaller space between the (needle) core and the shell, which increases the shear by the gas flow, results in smaller microspheres (Figure S2, Supporting Information, panel b). Generally, we can fabricate high‐quality microspheres using a receiving distance larger than 9 cm, a flow rate of the Na‐Alg solution between 2 and 8 mL h^−1^ and concentrations of Na‐Alg and CaCl_2_ ranging between 0.4–3.0% and 2.0–8.0%, respectively. To score the production throughput of the microspheres, we collected Na‐Alg droplets on A_4_ paper (Figure S3, Supporting Information), which indicated that about 2000 particles could be produced in only 20 s (at a pump speed of 3 mL h^−1^). We concluded that the throughput from the gas‐shearing method is at least competitive with microfluidic and centrifugation‐based methods reported on in previous studies.[[qv: 14c,23]]

**Figure 2:**
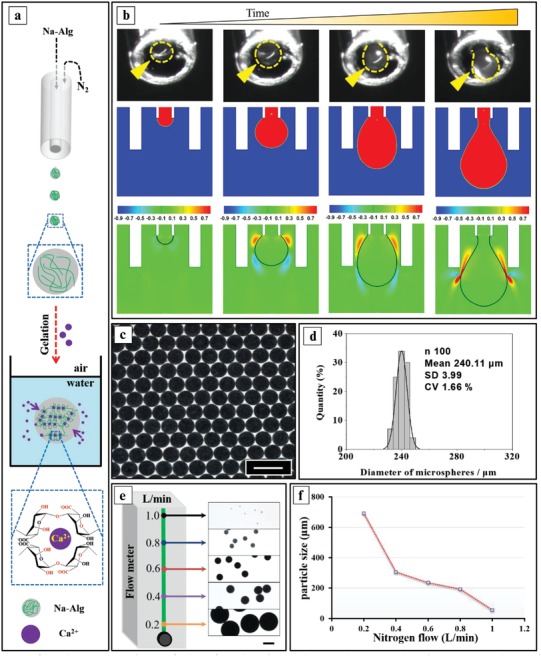
The generation of one‐faced microspheres by gas‐shearing. a) Schematic illustration of an SED‐1 generating isotropic microspheres and the crosslinking reaction of the Na‐Alg droplets by Ca^2+^ ions. b) Top panel: high‐speed snapshots of the droplet formation; the dashed yellow lines indicate the profiles of the droplet; Middle panel: CFD simulation of the droplet formation; Bottom panel: simulation of the velocity (m s^−1^) field. c) Optical image of the obtained microspheres loaded with Fe_3_O_4_ nanoparticles. d) Size distribution of the microspheres. e) Optical images of one‐faced microspheres fabricated at various nitrogen flow rates. f) The relationship between the particle size and the nitrogen flow. The scale bars in all panels are 400 µm.

As shown in **Figure** **3**, the SED‐2, SED‐4, SED‐6, and SED‐8 configurations were designed to evaluate if the gas‐shearing approach would allow MCMs. Interestingly, two‐, four‐, six‐, and eight‐faced MCMs could be successfully produced from Na‐Alg solutions containing green or red polystyrene nanospheres (200 nm), as shown in Figure 3 and Figures S4 and S5, Supporting Information. The fluorescence intensity profiles indicate that the various compartments within a single microparticle are physically well separated (Figure 3 and Figure S6, Supporting Information).

**Figure 3:**
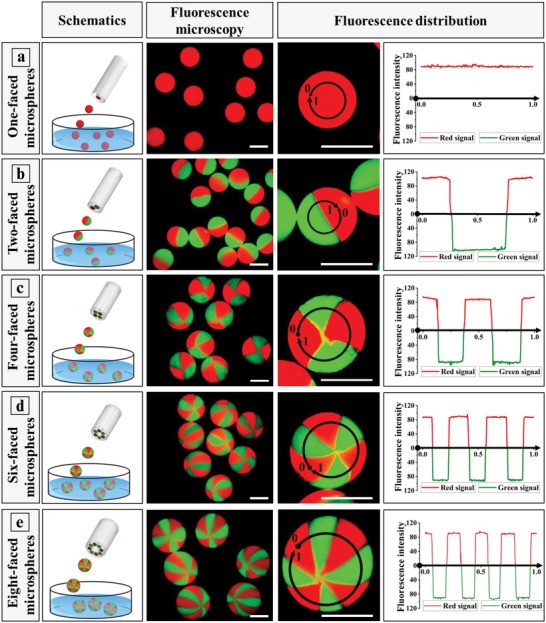
Generation of anisotropic multicompartmental microspheres, selectively loaded with green or red polystyrene nanospheres (200 nm) and produced by gas‐shearing of Na‐Alg solutions. The scale bars are 400 µm.

To interpret the versatility of the gas‐shearing approach, we tested if microspheres could be obtained from other water‐soluble polymers (such as chitosan; CS), and organic‐soluble polymers such as poly‐acrylonitrile (PAN), cellulose‐acetate (CA), ethyl‐cellulose (EC), poly‐caprolactone (PCL), cellulose‐acetate‐phthalate (CAP), and polyurethane (PU). As **Figure** **4**a illustrates, CS microsphere formation occurs through ionic crosslinking (such as for alginate microspheres), whereas for organic‐soluble polymers, solidification of the droplets occurs through the exchange of the dimethylformamide solvent with water (Figure 4b). Panel c in Figure 4 confirms the versatility of the gas‐shearing approach as various types of microparticles are easily obtained. Although the same fabrication conditions were used, the size of the microspheres was polymer dependent and could be ascribed to differences in viscosity and/or surface tension among these polymer solutions. It is interesting to observe that nanofibers can also be fabricated by simply increasing the nitrogen flow (Figure S7, Supporting Information).

**Figure 4:**
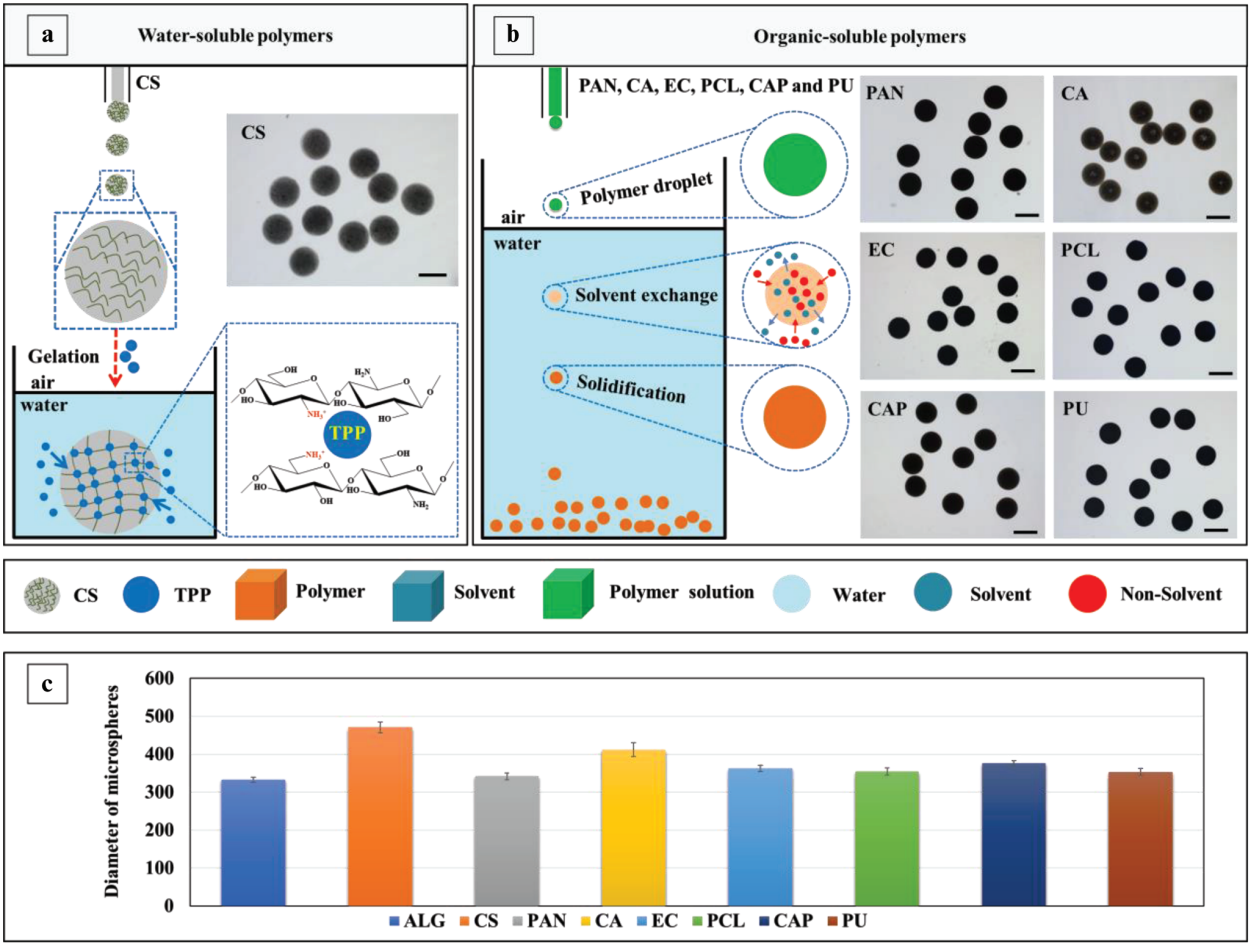
Versatility of gas‐shearing for the production of microspheres. Note that the SED, the nitrogen flow, and the flow rate of the polymer solution were identical in all the experiments. a) Schematic illustration of the ionic crosslinking (using thiamine pyrophosphate) of CS aqueous droplets into CS microspheres. b) Schematic illustration of the solvent exchange process resulting in PAN, CA, EC, PCL, CAP, and PU microparticles. c) The diameter of the obtained microspheres. The scale bars in panel a) and b) are 400 µm.

To illustrate the functionality of the multiple compartments in the MCMs, we took on the challenge of encapsulating magnetic Fe_2_O_3_ nanoparticles and cells in specific compartments of the MCMs. As shown in **Figure** **5**a and experimentally shown in Figure 5b, processing Fe_2_O_3_ containing Na‐Alg solutions through the needles of configuration SED‐8 allows fabrication of up to seven types of “asymmetric” (anisotropic) microspheres. As shown in Figure 5c‐iii and Movie S5, Supporting Information, the Ca‐Alg MCMs composed of alternating magnetic and nonmagnetic compartments can be rotated in a highly controlled manner using an external magnetic field. Such rotating “microrobots” may become of use in biomedicine and tissue engineering.

**Figure 5:**
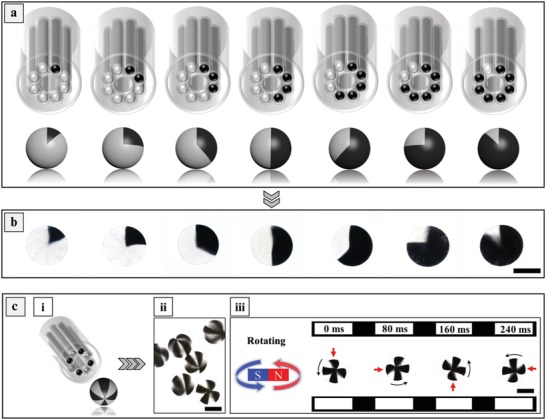
a) Schematic illustration of the fabrication process of anisotropic multifaced microspheres. b) Experimentally obtained anisotropic multifaced microspheres with various magnetic and nonmagnetic compartments. c) Schematic illustration of the fabrication process for eight‐faced symmetric microspheres (i). Microscope images of the eight‐faced symmetric microspheres (ii). Microscope images of the response of the eight‐faced microspheres in a rotating magnetic field (iii). The scale bars are 400 µm.

Currently, cell‐loaded gel microspheres are widely generated through microfluidics where water–oil phases are processed.^24^ However, harmful organic reagents are often used as the continuous phase.^25^ Although efforts have been undertaken to minimize the exposure time of the cell‐loaded microgels to these harsh conditions,^26^ cytotoxicity often remains a challenge. Therefore, for 3D cell culturing in microparticles, it is highly desirable to fabricate the microspheres in a simple high‐throughput way under oil‐free and surfactant‐free conditions. Therefore, we tested if the gas‐shearing approach allows mild encapsulation of cells in the various compartments of the MCMs. We first encapsulated HepG_2_ cells into Ca‐Alg microspheres, as shown in **Figure** **6**a‐i. The cells are well distributed in each microsphere (Figure 6a‐iii). To examine the biocompatibility of our method, we applied fluorescent staining to quantify the viability of cells in the microspheres. We observed cell clusters growing in the microparticles (Figure 6a‐iii,v) and found that after 1 day, 96% of the HepG_2_ cells in the microparticles were alive, which is superior to the cell survival measured using a nonaqueous processing system or other (such as the centrifugation‐based) methods.[[qv: 2b,22a,24a,27]] Even after 7 days, the cell viability remained as high as 91% (Figure 6a‐iv).

**Figure 6:**
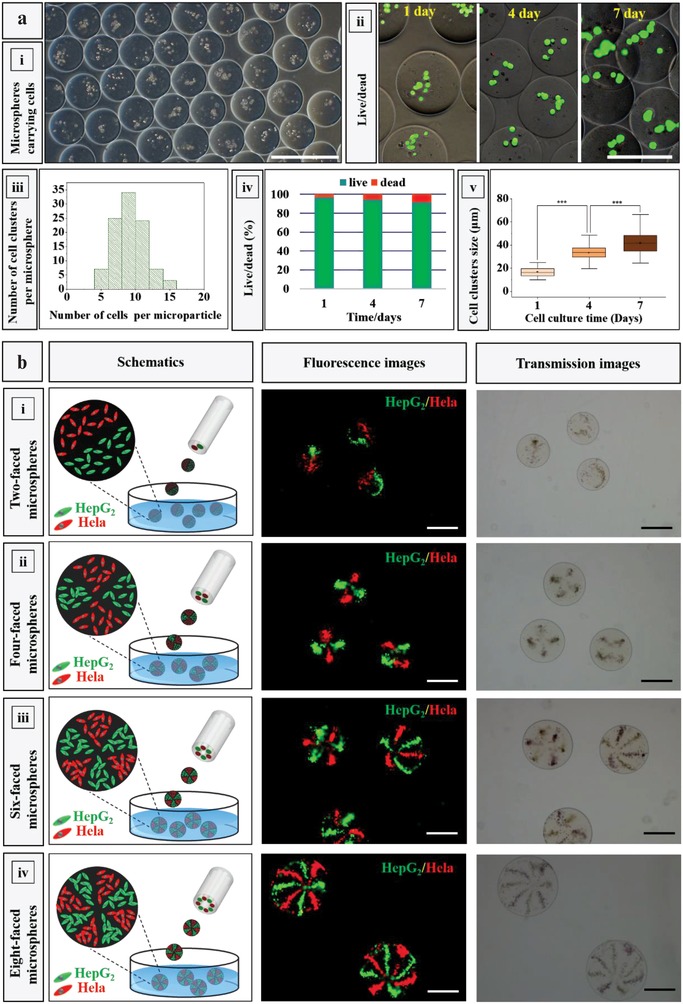
a) One‐step fabrication of the one‐faced microspheres carrying cells i) Ca‐Alg microspheres with encapsulated HepG_2_ cells. ii) Fluorescence microscopy images of the Ca‐Alg microspheres (the cells were stained with Calcein‐AM/PI) after 1, 4, and 7 days. iii) Number of cell clusters per microsphere. iv) Cell viability after 1, 4, and 7 days. v) Size of the cell clusters after 1, 4, and 7 days; ***p < 0.001. b) Multifaced Ca‐Alg microspheres carrying cells. The HepG_2_ and Hela cells were stained with DIO (green) and DiI (red), respectively. The scale bars in all images represent 400 µm.

Finally, we aimed to evaluate if the gas‐shearing method allows the fabrication of MCMs containing different cell types (HepG_2_ and Hela) in the various compartments of one single microparticle. Such highly controlled cell‐loaded MCMs with different cell types well separated from each other may be of interest for 3D cell coculturing. As shown in the left panel of Figure 6b and experimentally shown in the middle panel of Figure 6b and Figure S8, Supporting Information, HepG_2_ and Hela cells (stained with DIO and DiI) were encapsulated in the Ca‐Alg MCMs and arranged for coculturing. The HepG_2_ and Hela cells became well separated and ordered into complex geometries. These results indicate that our strategy can be easily used to obtain multiple microenvironments within a one single microparticle to precisely assemble different cell types within a confined micrometer‐sized volume. Note that, to our knowledge, the successful encapsulation of different cell types in eight‐faced MCMs has never been reported. We believe that the platform established here might provide an effective strategy to study the complex interactions between different cells. As optimal rheological properties of a cell matrix are needed to allow optimal cell growth, which may be cell type dependent,^28^ one can anticipate that MCMs composed of compartments with different viscoelastic properties can further add value to the materials investigated in this study.

In summary, a one‐step strategy, based on gas‐shearing, has been presented for the fabrication of MCMs composed of up to eight compartments. We show that this fabrication approach is highly versatile, as both aqueous and organic polymer solutions can be processed, whereas the morphology and size of the microspheres can be flexibly controlled using an appropriate SED and adjusting the gas flow. Our study suggests that the obtained MCMs may have highly versatile applications in bioengineering, especially as carriers for cells, which remains a key challenge to the progress of the field of tissue engineering.

## Conflict of Interest

The authors declare no conflict of interest.

## Supporting information

Supplementary

Supplementary

Supplementary

Supplementary

Supplementary

Supplementary
