# Supplementary material for: Gas‐Shearing Fabrication of Multicompartmental Microspheres: A One‐Step and Oil‐Free Approach
Source: Adv Sci (Weinh). 2019 Feb 28;6(9):1802342. doi: 10.1002/advs.201802342 (PMC6498303; doi:10.1002/advs.201802342)
Supplement: Supplementary file 1 — Supplementary [file ADVS-6-1802342-s002.pdf]

Copyright WILEY-VCH Verlag GmbH & Co. KGaA, 69469 Weinheim, Germany, 2019.

ADVANCED SCIENCE
Open Access

Supporting Information

for *Adv. Sci.,* DOI: 10.1002/advs.201802342

Gas-Shearing Fabrication of Multicompartmental Microspheres: A One-Step and Oil-Free Approach

Guosheng Tang, Ranhua Xiong, Dan Lv, Ronald X. Xu, Kevin Braeckmans, Chaobo Huang, and Stefaan C.De Smedt**

Copyright WILEY-VCH Verlag GmbH & Co. KGaA, 69469 Weinheim, Germany, 2019.

Supporting Information

Gas-Shearing Fabrication of Multicompartmental Microspheres: a One-Step and Oil-Free Approach

Guosheng Tang, Ranhua Xiong, Dan Lv, Ronald X. Xu, Kevin Braeckmans, Chaobo Huang and Stefaan C. De Smedt**

1. Experiment section

1.1 Materials

Sodium alginate (Na-Alg), Chitosan (CS, the degree of deacetylation is ≥95%), polyacrylonitrile (PAN, Mw: 88 000 g·mol^{-1}), Cellulose Acetate (CA), ethylcellulose (EC), polycaprolactone (PCL, Mw: 80 000 g·mol^{-1}), polyurethane (PU, Mw: 100 000 g·mol^{-1}) and Calcium chloride (anhydrous) were all purchased from Sinopharm Chemical Reagent Co., Ltd. (Shanghai, China). Cellulose-acetate-phthalate (CAP, Mw: 2 500 g·mol-1) was purchased from Sigma-Aldrich (Missouri, USA). Fluorescent polystyrene nanoparticles (200 nm), FL-PS-R-002 (excitation/emission: 620/680) and FL-PS-G-002 (488/518) were procured from DAE (Tianjin, China). Fe_3O_4 nanoparticles (30 nm) were purchased from Shanghai Macklin Biochemical Co., Ltd. (Shanghai, China). Float-type flowmeters (LAB-3WB, LZB-4WB, LZB-6WB) were purchased from Xiangyun flow meter factory (Shanghai, China). 3,3′-Dioctadecyloxacarbocyanine perchlorate (DiO), 1,1'-dioctadecyl-3,3,3',3'-tetramethylindocarbocyanine perchlorate (DiI) and the Calcein-AM/PI Double Stain Kit were purchased from Yessen (Shanghai, China). Dulbecco’s Modified Eagle’s medium (DMEM), fetal bovine serum (FBS), penicillin-streptomycin, Tryspin (0.25% Trypsin EDTA), and phosphate buffered saline (PBS) were acquired from Hyclone Laboratories (Logan, UT, USA). Water with a resistivity of 18.2 MΩ·cm^{-1} was acquired using a Millipore Milli-Q system. All

other chemical reagents were of the highest grade available and used as received. $HepG_2$ cells and Hela cells were obtained from the Cell Bank of the Chinese Academy of Sciences (Shanghai, China).

In all of the experiments, except for the section on special instructions, the collecting bath was 2% (w/v) calcium chloride aqueous solution, the pre-gel aqueous phases were 2% (w/v) sodium alginate aqueous solution with approximately 0.1% (w/v) fluorescent polystyrene nanoparticles, the collector receiving distance was 10 cm, the airflow was 0.6 L/min, the angle between the smart ejector and collector was 90°, and the flow rate of the Na-Alg was 3 ml/h. The solutions were all filtered before pumping into the Spray Ejector Device (SED). The airflow was set to 6 L/min for the generation of multicompartmental microspheres. Blunt needles and epoxy adhesive were purchased from the Taobao website based in China. All other chemical reagents were of the best grade available and used as received.

1.2 Equipment and assembly of the smart ejector devices (SEDs)

Equipment. As shown in Figure S1A, the equipment for fabrication of the microparticles through gas-shearing consists of four major parts: a digital injection pump (to pump the polymer (e.g., Na-Alg) solution), a collecting bath (either 2% (w/v) of $CaCl_2$ or water) to harvest the microparticles, a gas-holder (which provides the nitrogen flow, which is controlled by a rotameter) and a homemade Spray Ejector Device (SED; see below).

SEDs. As shown in Figure S1A, one-faced microspheres were obtained via a homemade SED-1 device. The SED-1 device consists of a ‘liquid-flow needle’ (30G), which is coaxially inserted in a ‘shell needle’ (20G or 18, 16, 14G). A 14G needle is used to transport the nitrogen. All the junctions were sealed using epoxy-adhesives. As Figure S1B shows, for SED-2/-4/-6/-8, the appropriate number (2, 4, 6 or 8) of liquid-flow needles was inserted in the shell needle. We note that for SED-6 and SED-8, an extra needle (the ‘holder needle’, 25G or 30G) was used to support the liquid-flow needles.

1.3 Fabrication of multicompartmental microspheres by gas-shearing

One-faced microspheres. One-faced microspheres were prepared by processing a Na-Alg solution through a SED-1 (30 G as the liquid-flow needle and a 20 G as the shell-needle). Since Ca-Alg microspheres are semitransparent, we added 0.2% (w/v) Fe_3O_4 nanoparticles (20 nm) to the Na-Alg solution. Experimental parameters such as the nitrogen flow, receiving angle, space between the core and shell, receiving distance, flow rate of the Na-Alg solution and concentration of the Na-Alg and $CaCl_2$ solution were varied, as indicated in the figures.

Multifaced microspheres. Multicompartmental microspheres were prepared in a similar way making use of SED-2, SED-4, SED-6 and SED-8. To visualize the various compartments in the microspheres (red and green), fluorescent polystyrene nanoparticles were added to the Na-Alg solution (the concentration of the polystyrene nanoparticles was about 0.1% (w/v)).

To fabricate the microspheres through the gas-shearing of polymers (other than alginate) dissolved in water or organic solvents, we used 0.3% (w/v) CS in aqueous solution, 10% (w/v) PU, 12% (w/v) PAN, 10% (w/v) CA, 25% (w/v) CAP and 40% (w/v) EC (all in DMF). The collecting bath was a 2% (w/v) sodium tripolyphosphate (TPP) solution in the case of CS solutions and water in the case of the organic polymer solutions.

Asymmetric multifaced microspheres. Asymmetric (magnetic) multicompartmental microspheres were obtained through the processing of a 2% (w/v) sodium alginate solution containing 0.2% (w/v) Fe_3O_4 nanoparticles through one or more liquid-flow needles of a SED-8.

Cell-loaded microspheres. One-faced and multifaced microparticles loaded with cells were obtained through the processing of a Na-Alg solution (1.5% (w/v)) containing cells (approximately 3.3×10^5 cells mL^{-1} to prepare one-faced microspheres and 3.3×10^6 cells mL^{-1} to prepare multicompartmental microspheres). All solutions and devices were sterilized before use. The produced microspheres were collected into culture medium (high sugar DMEM supplemented with 10% FBS and 1% penicillin-streptomycin) in cell culture plates and then incubated in the incubator (Thermo Forma 3111, Thermo, USA) using 5% CO_2 at 37 ℃.

During cell culturing, the cells were stained with 10 μM Calcein-AM/PI Double Stain Kit (Yessen, Shanghai, China) for observation. For the fabrication of the multicompartmental microspheres loaded with both $HepG_2$ and Hela cells, the cells were stained (with 20 μM DIO and 15 μM DII solutions, respectively) before dispersing them in the Na-Alg solution for further processing.

1.4 Characterization of the multicompartmental microspheres

The production of the one-faced microspheres (using the SED-1) was monitored in real-time using a microscope (C6230, Shanghai Zhong Chen Digital Technic Apparatus Co., Ltd.) and recorded by a high-speed camera (CCD, DH-MER-130-30UM). Bright-field and fluorescence images were snapped by microscopy (OLYMPUS IX53). Fluorescence photographs of the cross-sectional view of the multicompartmental microspheres were taken using a Laser Scanning Confocal Microscope (Carl Zeiss, LSM710). The microspheres and fibers were further characterized by a scanning electron microscope (FE-SEM, S-4800). Computational fluid dynamics (FLUENT (ANSYS 12.1)) was used to simulate the droplet dynamics. The droplet dynamics was tracked using the volume of fraction (VOF) model, which solves a system of momentum equations and treats the volume fraction of each fluid unit throughout the domain. More details about the VOF model can be found in our previous work [1].

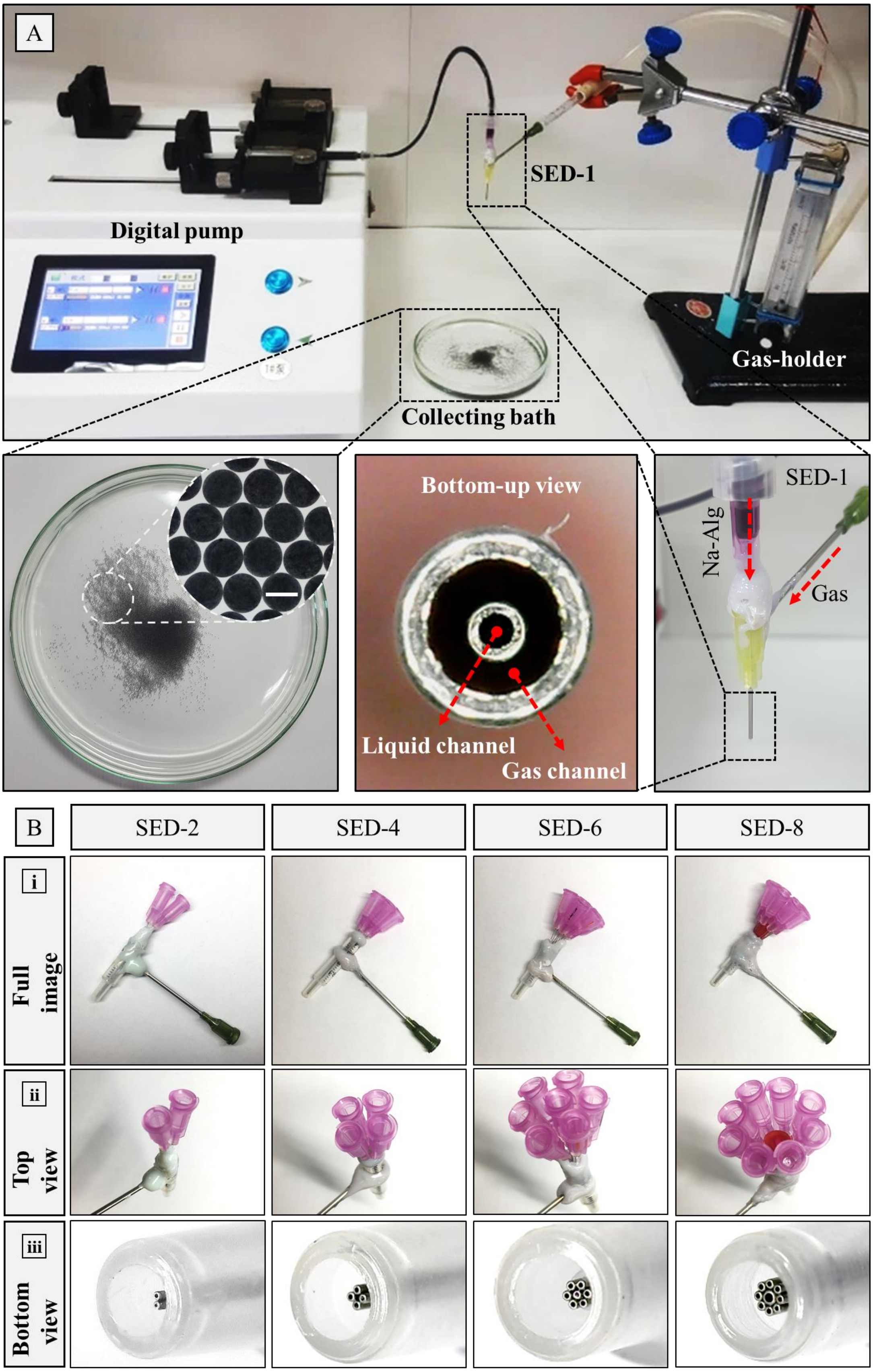

Figure S1. A) Images of the equipment to generate 'one-faced' microparticles. B) Photographs of the SEDs. Note that a central holder was added to optimally align the needles coaxially in the SED-6 and SED-8 (and to keep the needle system stable). The scale bar is 400 µm.

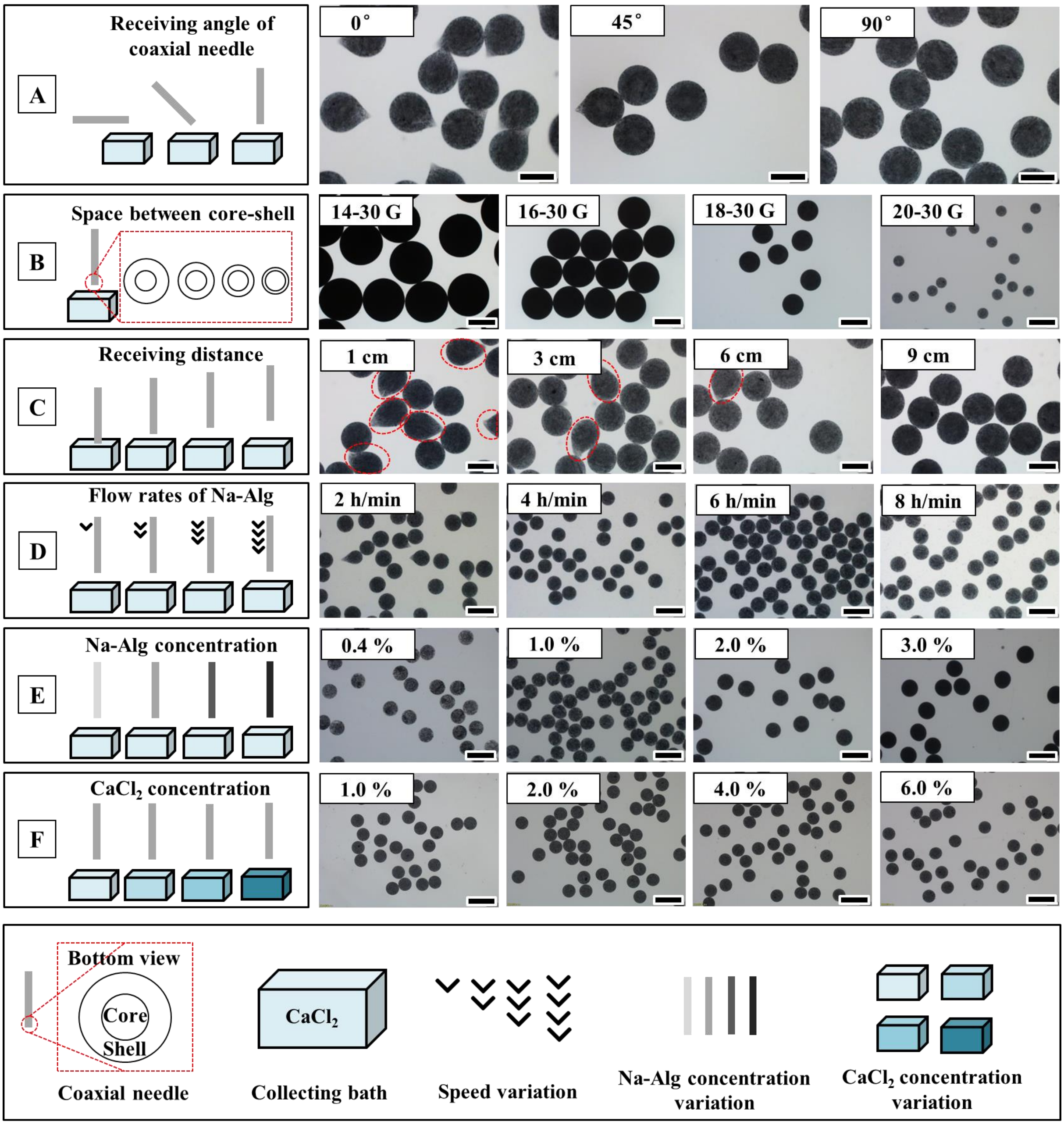

Figure S2. Microscope images of one-faced microspheres obtained under various experimental conditions. A-D) The influence of the receiving angle (A), space between the core and shell (B), receiving distance (C), flow rate of the Na-Alg solution (D), the Na-Alg concentration (E) and the $CaCl_2$ concentration in the collection bath (F). The scale bar is 400 μm.

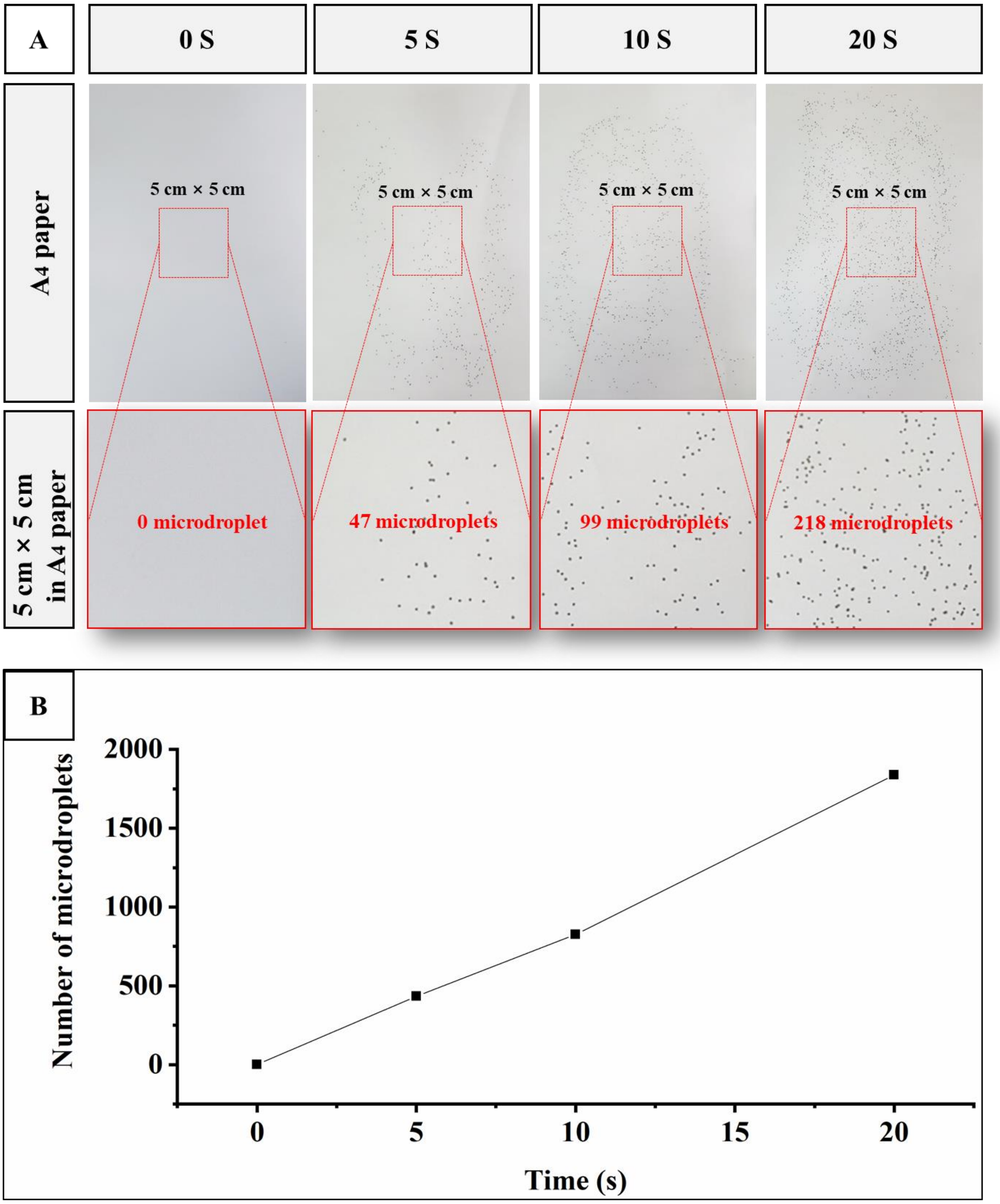

Figure S3. A) Na-Alg microdroplets as collected on A_4 paper after 0, 5, 10 and 20 s. B) The correlation between the number of microdroplets and the 'collection' time.

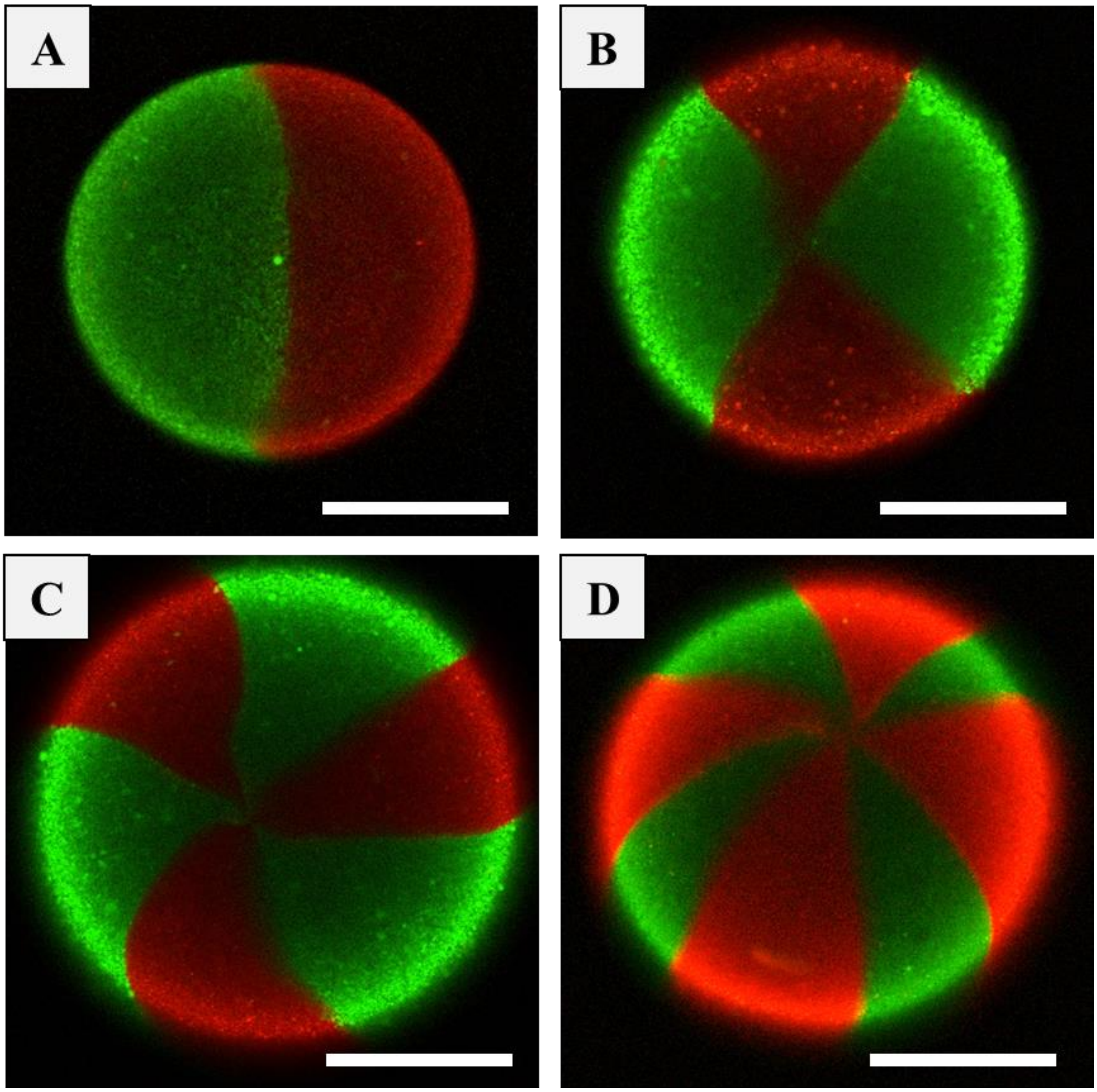

Figure S4. Confocal microscopy images of multicompartmental microspheres prepared from Na-Alg solutions. A-D) Confocal microscopy images of two-faced (A), four-faced (B), six-faced (C) and eight-faced (D) microspheres. The scale bars are 200 μm.

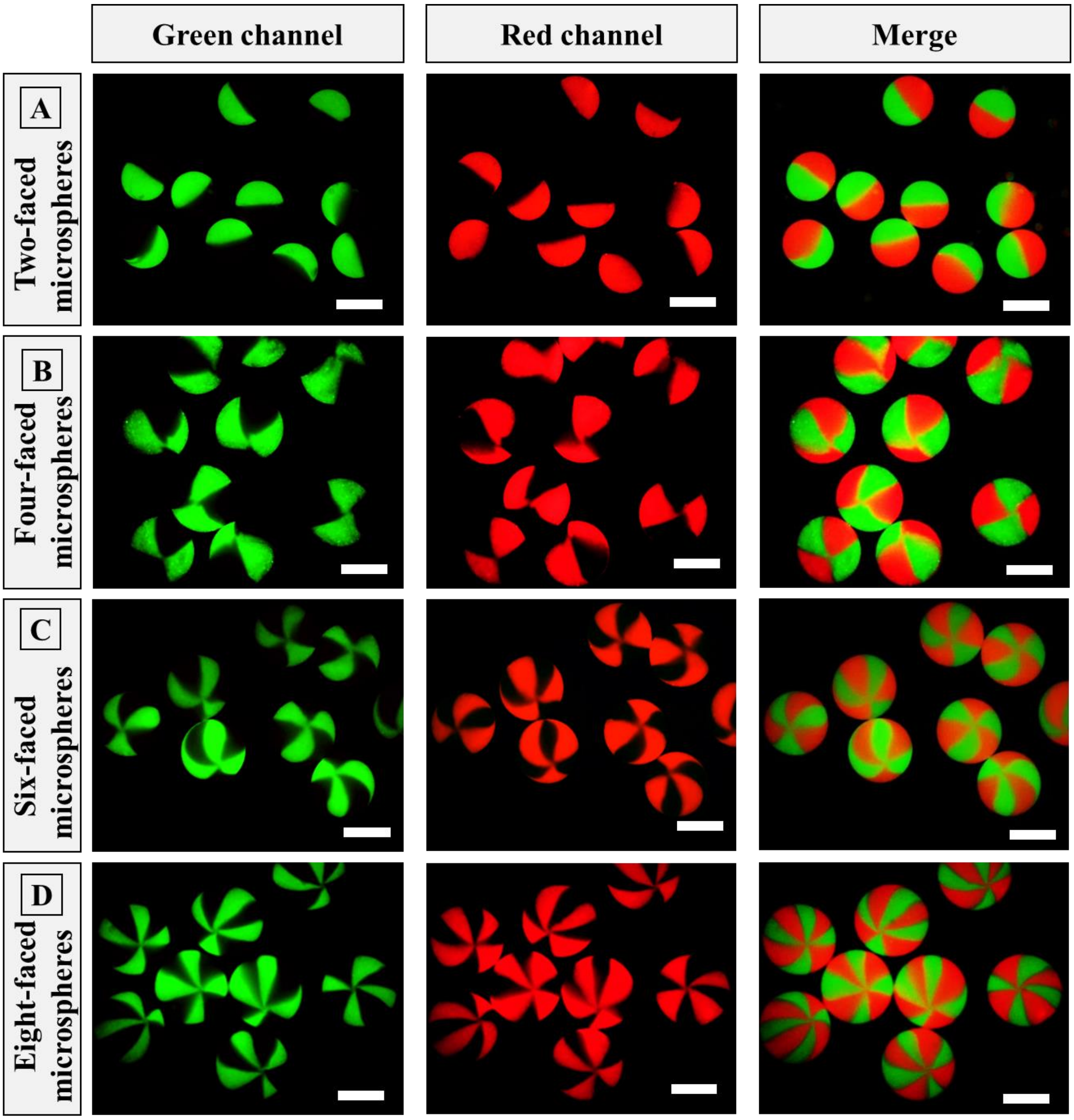

Figure S5. Red, green and merged fluorescence images of multicompartmental microspheres obtained from the Na-Alg solutions. The scale bar is 400 μm.

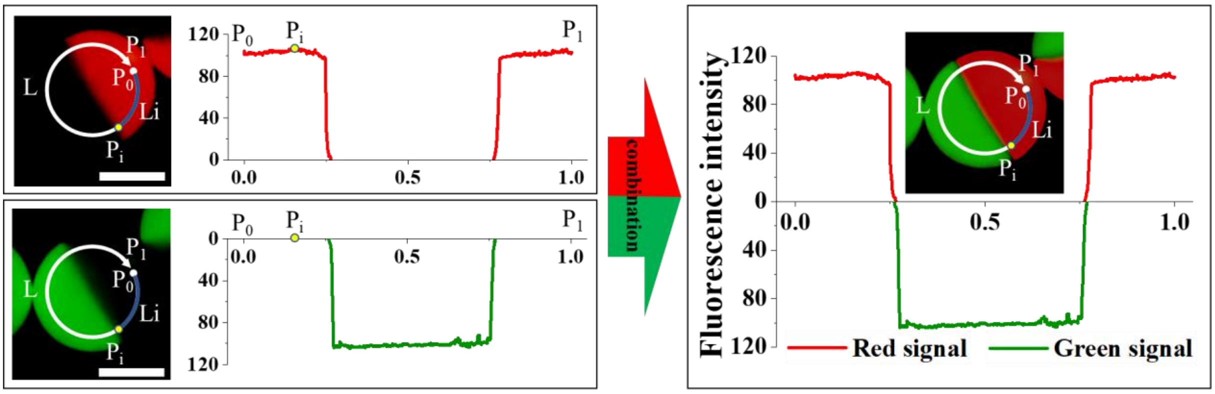

Figure S6. Fluorescence distribution (in two-faced microspheres in this example). For the circle, the starting point of the circle is 0 and the end point is 1.

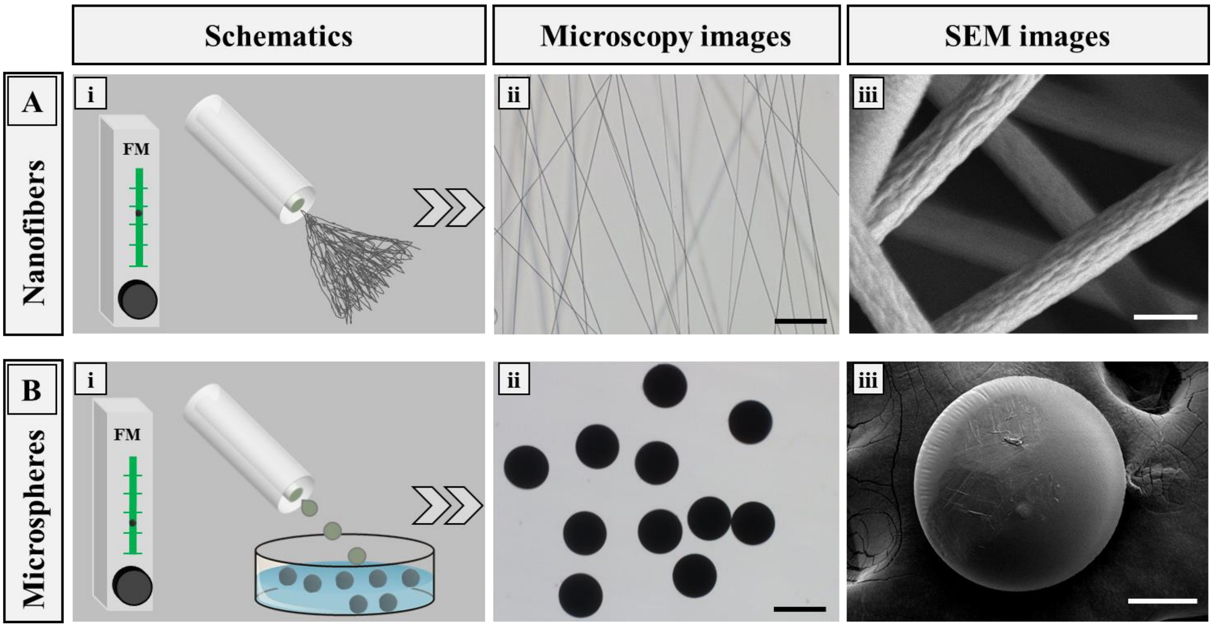

Figure S7. Formation of nanofibers and microspheres by gas-shearing of the PAN solutions. The scale bars are 100 μm in A- ⅱ, 1 μm in A- ⅲ, 400 μm in B- ⅱ and 100 μm in B- ⅲ.

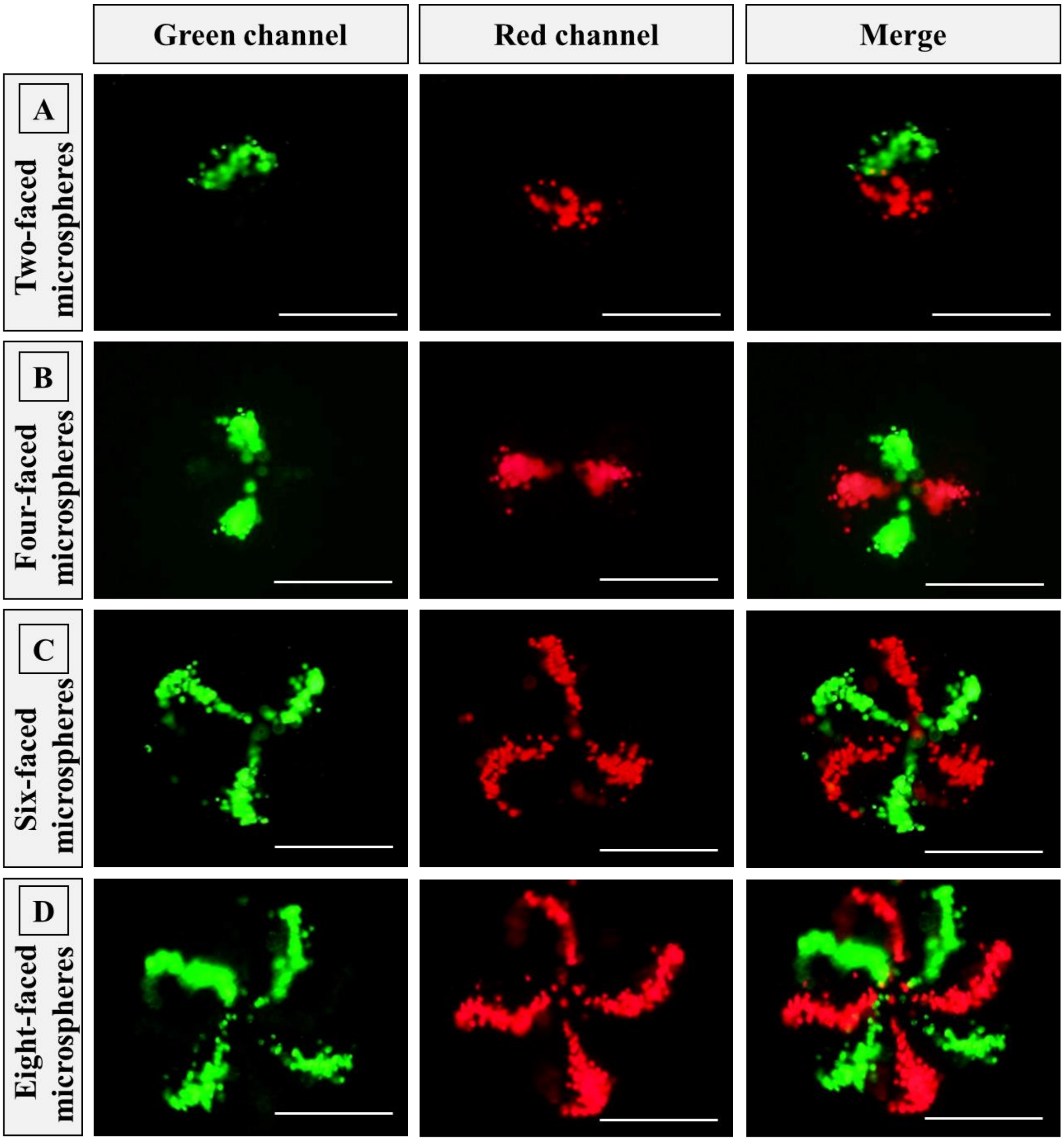

Figure S8. Red, green and merged fluorescence images of the multicompartmental microspheres with various encapsulated cell types. A-D) two-faced (A), four-faced (B), six-faced (C) and eight-faced (D) microspheres. Scale bar is 400 μm.

Table: Comparison of this work with other relevant literature

**Relevant work**		The power resources	With soft/green method	With eight-faced microspheres	With widely candidate polymers	With narrow size distribution	With large-scale adjustable size	With simple equipment and cost
This work		Gas-shearing	√	√	√	√	√	√
Centrifugation method	Takeuchi group (Adv. Mater, 2012; Small, 2015; Sci. Rep, 2016) [2]<br>Kim group (Chem. Mater, 2012; Acs. Appli. Mater. Interfaces, 2014)[3]<br>Xu group (J. Colloid Interf. Sci, 2016)[4]<br>Onoe group (Adv. Healthc. Mater, 2017)[5]	Centrifugal force	×	×	×	√	×	√
Microfluidic method	Nisisako Group (Chem. Eng. J, 2004; Adv. Mater, 2006; Adv. Mater, 2007)[6]<br>Kumacheva group (JACS, 2006)[7]<br>Weitz Group (Langmuir, 2009; Adv. Mater, 2009; Langmuir, 2010)[8]<br>Chen Group (Adv. Mater, 2011; Angew Chem Int Edit, 2012)[9]<br>Zhao group (JACS, 2013; Nanoscale, 2013)[10]	Oil cut	×	×	√	√	√	×
Electrified co-jetting method	Lahann group (Nat Mater, 2005; JACS, 2006; Adv. Mater, 2012; PNAS, 2012; Adv. Healthc. Mater, 2016)[11]<br>Shum group (Biomicrofluidics, 2013)[12]<br>Lai group (Acs. Appli. Mater. Interfaces, 2014)[13]	High voltage	×	×	√	×	√	×

2. Supporting Movies

Movie S1. The device for fabrication of the one-faced microspheres.

Movie S2. The generation of one-faced microspheres in the collecting bath.

Movie S3. Droplet formation dynamics-CFD simulation.

Movie S4. Droplet formation dynamics-Experimental movie.

Movie S5. Response of the magnetic eight-faced microspheres under the influence of a rotating magnet.

Reference

[1] R. Xiong, A. Y. Nikiforov, P. Vanraes, C. Leys, *Physics of Plasmas* **2012**, 19, 236.

[2] a) K. Maeda, H. Onoe, M. Takinoue, S. Takeuchi, *Advanced materials* **2012**, 24, 1340; b) S. Habasaki, W. C. Lee, S. Yoshida, S. Takeuchi, *Small* **2015**, 11, 6391; c) M. Hayakawa, H. Onoe, K. H. Nagai, M. Takinoue, *Scientific reports* **2016**, 6, 20793; d) Y. Morimoto, M. Onuki, S. Takeuchi, *Advanced healthcare materials* **2017**, 6, 1601375.

[3] a) D. H. Kang, H. S. Jung, N. Ahn, S. M. Yang, S. Seo, K. Y. Suh, P. S. Chang, N. L. Jeon, J. Kim, K. Kim, *ACS applied materials & interfaces* **2014**, 6, 10631; b) J. Lee, J. Kim, *Chemistry of Materials* **2012**, 24, 2817.

[4] M. Liu, X. T. Sun, C. G. Yang, Z. R. Xu, *Journal of colloid and interface science* **2016**, 466, 20.

[5] S. Yoshida, M. Takinoue, H. Onoe, *Advanced healthcare materials* **2017**, 6, 1601463.

[6] a) T. Nisisako, T. Torii, T. Takahashi, Y. Takizawa, *Advanced materials* **2006**, 18, 1152; b) T. Nisisako, T. Torii, T. Higuchi, *Chemical Engineering Journal* **2004**, 101, 23; c) T. Nisisako, T. Torii, *Advanced materials* **2007**, 19, 1489.

[7] Z. Nie, W. Li, M. Seo, S. Xu, E. Kumacheva, *Journal of the American Chemical Society* **2006**, 128, 9408.

[8] a) S. Seiffert, M. B. Romanowsky, D. A. Weitz, *Langmuir* **2010**, 26, 14842; b) R. K. Shah, J.-W. Kim, D. A. Weitz, *Advanced materials* **2009**, 21, 1949; c) C. H. Chen, R. K. Shah, A. R. Abate, D. A. Weitz, *Langmuir* **2009**, 25, 4320.

[9] a) S. N. Yin, C. F. Wang, Z. Y. Yu, J. Wang, S. S. Liu, S. Chen, *Advanced materials* **2011**, 23, 2915; b) Z. Yu, C. F. Wang, L. Ling, L. Chen, S. Chen, *Angewandte Chemie* **2012**, 51, 2375.

[10] a) Y. Zhao, H. Gu, Z. Xie, H. C. Shum, B. Wang, Z. Gu, *Journal of the American Chemical Society* **2013**, 135, 54; b) L. Shang, F. Shangguan, Y. Cheng, J. Lu, Z. Xie,

Y. Zhao, Z. Gu, *Nanoscale* **2013**, 5, 9553.

[11] a) K. H. Roh, D. C. Martin, J. Lahann, *Nature materials* **2005**, 4, 759; b) K. H. Roh, D. C. M. And, J. Lahann, *Journal of the American Chemical Society* **2006**, 128, 6796; c) A. C. Misra, S. Bhaskar, N. Clay, J. Lahann, *Advanced materials* **2012**, 24, 3850; d) K. J. Lee, J. Yoon, S. Rahmani, S. Hwang, S. Bhaskar, S. Mitragotri, J. Lahann, *Proceedings of the National Academy of Sciences* **2012**, 109, 16057; e) S. Rahmani, A. M. Ross, T. H. Park, H. Durmaz, A. F. Dishman, D. M. Prieskorn, N. Jones, R. A. Altschuler, J. Lahann, *Advanced healthcare materials* **2016**, 5, 94.

[12] Z. Liu, H. C. Shum, *Biomicrofluidics* **2013**, 7, 44117.

[13] W. F. Lai, A. S. Susha, A. L. Rogach, *ACS applied materials & interfaces* **2016**, 8, 871.
